# The structural and functional aspects of exercise-induced cardiac remodeling and the impact of exercise on cardiovascular outcomes

**DOI:** 10.1080/07853890.2025.2499959

**Published:** 2025-05-16

**Authors:** Natalie A. Van Ochten, Emmett Suckow, Lindsay Forbes, William K. Cornwell

**Affiliations:** ^a^ Department of Medicine, University of Colorado Anschutz Medical Campus, Aurora, Colorado, USA; ^b^ Department of Medicine-Cardiology, University of Colorado Anschutz Medical Campus, Aurora, Colorado, USA; ^c^ Clinical Translational Research Center, University of Colorado Anschutz Medical Campus, Aurora, Colorado, USA; ^d^ Department of Medicine-Pulmonary and Critical Care Sciences, University of Colorado Anschutz Medical Campus, Aurora, Colorado, USA

**Keywords:** Exercise guidelines, cardiovascular disease, exercise-induced cardiac remodeling

## Abstract

The relationship between exercise training and overall cardiovascular health is well defined, with associated reductions in incidence of cardiovascular disease and comorbidities such as hypertension, diabetes mellitus, cholesterol profile, body weight, and even some forms of cancer. In this regard, an exercise prescription is an effective tool to decrease all-cause mortality and improve overall health. As providers, properly educating patients on the type, amount, and intensity of exercise is important to ensure patients meet recommended exercise metrics to experience these health benefits. This review provides a concise, but comprehensive overview of the structural and functional aspects of exercise-induced cardiac remodeling, current recommended guidelines for exercise with supporting data, as well as the impact exercise has on various cardiovascular outcomes.

## Introduction

Ischemic heart disease and stroke are the number one and number two causes of death worldwide, respectively [1]. Lifelong adherence to a routine exercise program leads to several cardiometabolic improvements, including cholesterol profile, glucose control, blood pressure (BP) control and endothelial function. In addition, exercise training is associated with reductions in hard outcomes including acute myocardial infarction (MI), stroke, all-cause mortality, and even some cause-specific mortalities such as death due to CVD and some forms of cancer. Major health agencies throughout the United States of America, such as the Department of Health and Human Services, and the American Heart Association (AHA), have developed guidelines to provide clinicians and patients with information on an optimal dose of exercise to improve overall health. This review provides a comprehensive overview of the impact of exercise on cardiovascular structure and function, metabolic benefits of exercise, as well as data supporting exercise guidelines.

## Exercise-induced cardiac remodeling

**Figure 1.**
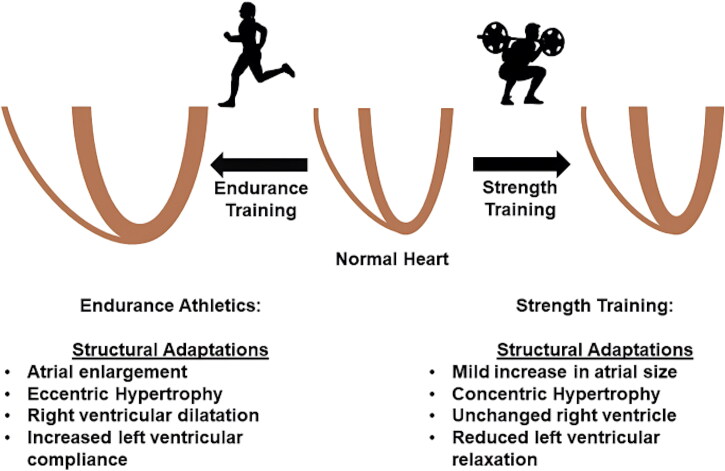
Model of exercise-induced cardiac remodeling. While overlap exists between different types of sport, highly dynamic exercise training generally imposes a volume load on the heart, leading to eccentric hypertrophy of the ventricles and atrial enlargement. Strength training (static exercises) impose a pressure load on the heart, leading to concentric hypertrophy and in some cases, impaired ventricular relaxation. Image reproduced from senior author with permission [2].

Exercise-induced cardiac remodeling (EICR) describes cardiovascular structural, functional, and electrical remodeling that occurs in response to regular intense exercise (Figure 1) [3,4]. The specific cardiac size and structure can vary depending on the type, intensity, and duration of exercise [5,6]. Generally, exercise is divided into two broad categories: static and dynamic. Static exercise refers to the intensity of static muscle contraction undertaken as a proportion of an individual’s maximal voluntary contraction. The dynamic component is determined by the oxygen uptake (VO_2_) required to engage in any specific exercise. Static exercises increase the pressure load, while dynamic exercises increase volume load and cardiac output (Qc).

The term “athlete” does not have a standard definition. Generally, “athlete” is applied to individuals engaging in any number of sporting disciplines based on the level of dynamic and static load with varying levels of duration/intensity of their respective sport, including elite and recreationally active individuals [1,7]. Recognizing the heterogeneity in the definition, there have been attempts to standardize the definition of athlete according to the intent of exercise (e.g. personal vs. recreational vs. competition), the volume of exercise (hours/week) and level of competition [8]. Along these lines, there is no single phenotype that describes EICR in athletes. Rather, EICR varies in accordance with the degree of dynamic vs. static exercise, recognizing that almost all exercises have varying degrees of both.

### Dynamic versus static exercise

**Figure 2.**
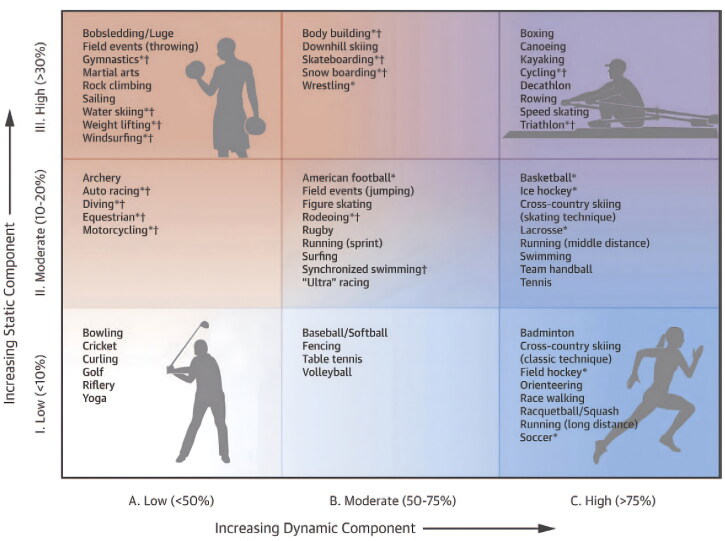
The classification of different sports/exercises is based on the amount of static vs. dynamic intensity is present. Image is borrowed from Levine et al. [10] with permission.

Dynamic exercises generally lead to eccentric hypertrophy, characterized by increased left ventricular end-diastolic volume (LVEDV) and cardiac mass [9]. These adaptations result from repetitive exposure to increased Qc and decreased total peripheral resistance (TPR) [9]. Examples of dynamic exercise include distance running, cross-country skiing, and soccer. Static exercise leads to concentric hypertrophy characterized by increased left ventricular (LV) wall thickness and cardiac mass [9]. These adaptations result from repetitive exposure to increased TPR [7,9]. Examples of static exercise include weight lifting, gymnastics, and rock climbing. In reality, the majority of exercises have varying degrees of both static and dynamic components, with overlap in the type and degree of cardiac remodeling that occurs [7]. Specific examples of dynamic vs. static exercise and various contributions of each are outlined in Figure 2 [7].

In a meta-analysis of several studies examining cardiovascular structure and function among athletes, when compared to controls, LV mass was 64% greater in cyclists, 48% greater in runners, and 25% greater in athletes who participated in regular strength training [11]. Another meta-analysis stratified athletes (*N* = 1451) into purely dynamic (running), purely static (weight lifting, wrestling), or combined (rowing, cycling) exercise and found that LV wall thickness in non-athletic controls was 0.36 mm, significantly smaller than athletes engaging in dynamic (0.39 mm), static (0.44 mm), or combined exercise (0.40 mm) [12]. Additionally, the combined exercise group had the largest cardiac mass (∼288 g) compared to the static (∼267 g), dynamic (∼249 g), and non-athletic controls (174 g) [12].

### Changes in the right heart

The right ventricle (RV) also undergoes structural and functional changes in response to exercise training [,^2^,^13–16^]. In an imaging analysis of elite male endurance athletes (*N* = 127) engaged in high-performance orienteering, middle-distance running, and cross-country skiing, compared to controls, athletes had greater right atrial (RA) and RV inflow tract sizes, as well as a greater RV free wall thickness as a result of increased hemodynamic loading from sustaining high cardiac outputs during exercise [17]. EICR variably impacts RV size depending on the sport [13,^2^,^18^,^19^]. For example, in an echocardiographic analysis of collegiate athletes engaged in rowing (*N* = 40) vs. football (*N* = 40), compared to baseline images, three months of training led to an increase in RV end-diastolic area among rowers (pre vs. post: 1,460 ± 220 mm/m^2^ vs. 1650 ± 200 mm/m^2^), while there was no change in RV end-diastolic area among the football players (1,340 ± 180 mm/m^2^ vs. 1,330 ± 120 mm/m^2^) [19].

### Ventricular lusitropy

**Figure 3.**
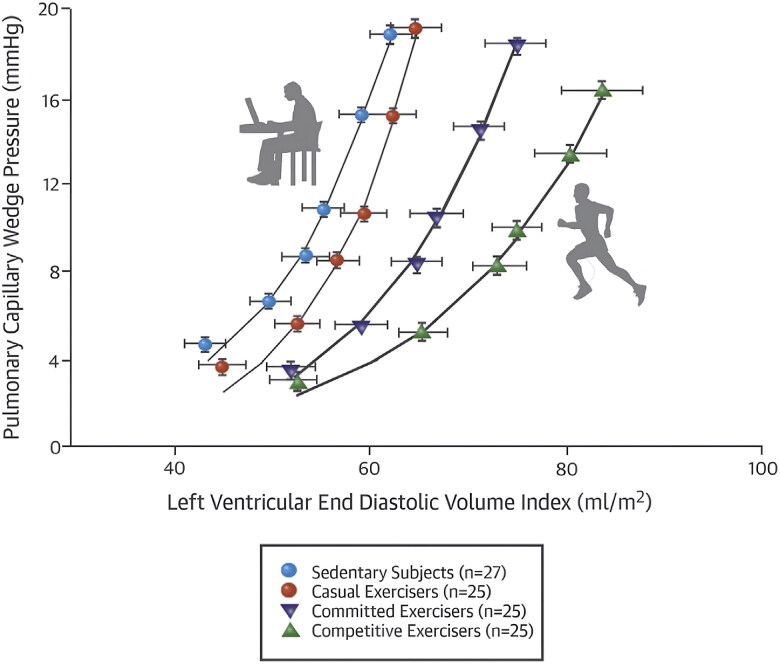
Left ventricular end-diastolic pressure versus volume relationship based on invasive hemodynamic assessment and echocardiogram analysis. This image shows the increased compliance in competitive exercisers (6–7 sessions/week throughout adult life) and committed exercisers (4–5 sessions/week) compared to casual exercisers (2–3 sessions/week) and sedentary individuals. Borrowed from bhella et al. [18] with permission.

Exercise positively impacts ventricular lusitropy- the rate of myocardial relaxation during diastole. This concept was elegantly demonstrated in a cross-sectional analysis of 102 individuals ages 67–70 years old who were stratified into one of four groups according to lifelong patterns of exercise: “Sedentary” (less than 2 exercise sessions/week), “casual” (2–3 sessions/week), “committed” (4-5 sessions/week), or “competitive” (6–7 sessions/week) [18]. LV end-diastolic pressure-volume curves were created using a combination of echocardiogram and invasive hemodynamics. LV compliance was greatest in the committed and competitive groups, whereas individuals who were sedentary throughout the adult lifespan had an up- and leftward shift in the LV end-diastolic pressure-volume curve consistent with an increase in LV stiffness (Figure 3) [18]. These data underscore the cardiovascular benefits associated with a lifelong commitment to exercise. Specifically, routine adherence to exercise, at least 4–5 times per week throughout adulthood, preserves ventricular compliance in the elderly years [18].

However, ventricular compliance may be restored among previously sedentary individuals who would otherwise experience age-related ventricular stiffening. Among previously-sedentary individuals (*N* = 53, age 53 ± 5 years) randomized to two years of exercise training versus yoga as a control, exercise led to an improvement in ventricular compliance, as demonstrated by a down and right-ward shift in the LVEDV curve [20]. There was no improvement in ventricular compliance among individuals randomized to yoga. The training protocol consisted of 5–6 h of exercise per week, incorporating a combination of high-intensity interval training, as well as short (30 min) and longer (60 min) base pace sessions. These data demonstrate that the heart has a high degree of plasticity, with an ability to reverse age-related stiffening that otherwise occurs as a result of sedentary aging, at least among otherwise healthy but sedentary individuals well into middle-age range (average age of individuals in this study was 53 ± 5 years) [20].

### Effect of exercise training on blood pressure

Acutely, BP increases in response to exercise, though the determinants of BP vary according to the type of exercise undertaken [1,21]. When considering the impact of exercise types on BP, it is helpful to recall the equation of mean arterial pressure (MAP):

MAP= Qc ×TPR,

where Qc is cardiac output and TPR is total peripheral resistance. During dynamic exercise, there is a large increase in Qc and a reduction in TPR. Peripheral vasodilatation results from this reduction in TPR and is necessary for the delivery of oxygenated blood to working muscle to generate adenosine triphosphate and sustain exercise. The increase in MAP during dynamic exercise is a result of the large increase in Qc. In normal individuals, Qc can increase by three- to five-fold, though among elite athletes this increase in Qc may be even greater [22]. During static exercise, however, there is a substantial rise in TPR as a result of the external load placed on the human body, combined with contraction of large skeletal muscle groups. The result is vasoconstriction with minimal change in Qc (at least when compared to the change in Qc during dynamic exercise) and a large increase in MAP. The increase in MAP is in proportion to the load placed on the body during static exercise. For example, it has been demonstrated that BP may increase to greater than 300-400 mmHg when completing weightlifting at, or near, a one-rep maximum load [23].

Hypertension has long been recognized as one of several major risk factors for cardiovascular disease, stroke, and atrial fibrillation. The protective effect of exercise is mediated, in large part, through this reduction in BP. Routine adherence to exercise training is associated with reductions in BP over time. In a ­systematic review and meta-analysis of randomized controlled trials (*N* = 9, total of 223 participants), 4 weeks or more of isometric exercise lowered systolic blood pressure (SBPmean reduction6.77 mmHg (*p* < 0.001), diastolic blood pressure (DBP, mean reduction: 3.96 mmHg (*p* < 0.001), and mean arterial pressure (MAP, -mean reduction: 3.94 mmHg (*p* < 0.001) [24].

In a systematic review and meta-analysis of 37 studies (*N* = 847 participants), exercise training led to a reduction in daytime SBPand DBP by sdfzxc4.06 (2.93–5.09 mmHg) and 2.77 (1.97–3.58 mmHg), respectively [25]. In this meta-analysis, the magnitude of reduction in BP was greater for patients with a baseline BP of greater 130/80 mmHg. Further, neither exercise intensity, duration of exercise program (median 12 weeks, range 1–78 weeks), nor total number of exercise sessions (median 39 sessions, range 6–156) impacted the magnitude of the reduction in BP associated with exercise [25]. Additional benefits of exercise that may favorably impact BP include improved endothelial function with increased nitric oxide production [26], as well as reductions in sympathetic nerve activity and inflammatory markers, and maintenance of arterial compliance [27–29].

### Cholesterol profiles

Endurance training has been associated with higher levels of circulating high-density lipoprotein (HDL) and a reduction in total triglycerides levels [30], favorable responses that are associated with reductions in risk of CVD [31]. In a study of 111 sedentary individuals with mild-to-moderate dyslipidemia, individuals were randomized into a control group vs. an 8-month exercise group of varying degrees of intensity: High amount and high intensity (23 kcal/kg/week, jogging), low amount and high intensity (14 kcal/kg/week, jogging), and low amount and moderate intensity (14 kcal/kg/week, walking) [32]. In the high-amount and high-intensity, low-amount and high-intensity, and low-amount and moderate-intensity groups, there were improvements in triglyceride concentration (*p* = 0.006, *p* = 0.07, and *p* < 0.001, respectively), concentration of VLDL triglycerides (*p* = 0.004, *p* = 0.04, and *p* < 0.001), and concentration of large VLDL particles (*p* = 0.05, *p* = 0.13, and *p* < 0.001). High-amount and high-intensity exercise had a larger effect on 10 of the 11 studied variables than low-amount and high-intensity exercise (P values between groups all <0.05). However, the low-amount and high-intensity exercise still had a beneficial effect on all 11 cholesterol variables analyzed compared to the control group [32].

In another analysis, investigators completed two randomized-control trials involving six different exercise groups of various exercise intensities and doses [33]. One of these trials assigned adults (*N* = 175) to 1 of 4 groups: low-amount/moderate-intensity exercise, high-amount/moderate-intensity exercise, high-amount/vigorous-intensity exercise, or clinical lifestyle intervention (diet and exercise). The other trial randomized individuals (*N* = 198) to a control group or 1 of 2 exercise groups that reflect the goals of overall health vs. weight loss. Combined results from these trials were that patients had significant increases in HDL levels with high-intensity and high-amount groups, but not in other groups [34]. In summary, exercise of any intensity and duration appears to favorably impact cholesterol profiles, though the response may be blunted among individuals engaged in mild-moderate exercise intensities.

## Impact of exercise training on survival and cardiovascular outcomes

The hemodynamic, physiologic and biochemical responses to exercise, as described, form the basis for the impact of exercise training on clinical outcomes.

Adherence to an exercise program is associated with reductions in all-cause and CVD-related mortality [34]. The AHA has recognized the centrality of cardiorespiratory fitness in promoting overall health and recommends assessing fitness for establishing a patient’s risk of mortality and CVD [35].

### Stroke risk

During an acute bout of exercise, there is an increase in risk of stroke during or immediately afterwords [1]. The Stroke Onset Study reported several informative pieces of information in this regard [36]. First, there is an approximate two-fold increase in risk of ischemic stroke within an hour of completing moderate-vigorous physical activity. However, this risk was greater among sedentary individuals, defined as those who exercise less than three times week, compared to those who exercise three or more times a week. In addition, the risk of ischemic stroke was approximately 2.5 times higher when lifting weights heavy weights (defined as 50 lbs or more) compared to exercise without heavy lifting [36]. In the ACROSS (Australasian Cooperative Research on Subarachnoid Hemorrhage Study), moderate-extreme exercise, defined as ≥5 metabolic equivalents, was associated with a three-fold increase in risk of subarachnoid hemorrhage within two hours of completing the exercise bout [37]. Importantly, this risk of subarachnoid hemorrhage was not attenuated by habitual exercise [37]. These observations are similar to other analyses demonstrating an increase in risk of myocardial infarction shortly following moderate-extreme exercise [38–41].

However, routine exercise is associated with long-term reductions in risk of stroke. In the REGARDS study (Reasons for Geographic and Racial Differences in Stroke), routine exercise of at least four session per week was associated with a reduction in stroke or transient ischemic attack over an approximate six-year period of follow-up [42]. However, in this study, the association between stroke and physical activity was partially attenuated after adjustment for traditional stroke risk factors (e.g. hypertension, diabetes), suggesting that the reduction in stroke risk is related, at least in part, from an interaction between routine exercise and attenuation of typical stroke risk factors [1].

### CVD and myocardial infarction

Similar to stroke, routine exercise reduces the risk of CVD over time, but there is an increased risk of myocardial infarction (MI) and sudden cardiac death in the short-term [38–41]. In an observational study of patients (*N* = 1228) interviewed within four days of an acute MI, 4.4% reported heavy exertion within one hour of symptom onset [38]. The relative risk of MI within one hour of heavy exercise was 5.9 (95% confidence interval 4.6–7.7) times the risk associated with less-strenuous exercise or no exercise, and the risk was greatest among individuals who were habitually sedentary [38]. Said another way, routine exercise may exert a protective effect over acute MI immediately following heavy exertion. Similar trends have been observed for sudden cardiac death. In an analysis of male physicians (*N* = 21,481) free of CVD in the Physicians’ Health Study, habitual vigorous exercise was associated with a reduction in risk of sudden death during vigorous exercise [41]. Benefits of exercise training have been observed on a molecular level. For example, among patients with stable CVD (*N* = 17) four weeks of exercise training with rowing or cycling improved endothelial nitric oxide synthase activity, as well as endothelium-dependent vasodilation [43].

### Sudden cardiac death and lay responder cardiopulmonary resuscitation

Much attention has been paid to sudden cardiac death (SCD) among recreational and professional athletes. Paul Thompson’s classic paper demonstrated that among joggers in Rhode Island, the incidence of SCD was 1 out of every 3,000,000 jogging-hours [44]. In a large analysis of 10.9 million runners completing long-distance races in the USA between 2000 and 2010, 59 had SCD (incidence 0.54 per 100,000 participants) [45]. The incidence of SCD was higher during marathons than half-marathons and was also greater among men than women [45]. Other analyses have demonstrated that the risk of SCD may be up to five-fold higher among black vs. white athletes across all ages and both sexes [46]. Causes of SCD are age-dependent [46]. For example, among younger individuals, genetic or congenital heart diseases are more common, while ischemic heart disease is the primary cause of death among Masters athletes (athletes 35 years of age or greater) [46].

The AHA and American College of Sports Medicine endorse preparticipation screening to identify athletes at risk for SCD [47]. Core aspects of the preparticipation screening assessment include a focused history to evaluate for symptoms, relevant family history and abnormalities on physical examination. Twelve-lead electrocardiogram improves sensitivity for detection of conduction abnormalities increasing the risk of SCD such as accessory pathways, channelopathies or heritable cardiomyopathies, but cannot reliably detect other structural abnormalities such as anomalous coronary arteries, aortopathies, or valvular abnormalities. There are conflicting data on whether the addition of ECG to preparticipation screening reduces risk above and beyond history and physical examination alone [48]. There are no data demonstrating that screening for coronary artery disease in asymptomatic individuals reduces mortality in the general population. However, guidelines from several national and international organizations recommend preparticipation screening with twelve-lead ECG and exercise stress testing for Masters athletes with one or more risk factors for cardiovascular disease [46].

Irrespective of the approach implemented to screen athletes, appropriate resources must be in place to mitigate risk when abnormalities are identified or when SCD occurs during sport. An effective emergency action plan depends on immediate recognition of an SCD event, implementation of high-quality cardiopulmonary resuscitation and access to an automated external defibrillator. School-based programs with effective emergency action plans provide a high survival rate for both student athlete as well as nonstudents who suffer SCD on school grounds [.

## Exercise guidelines and impact on outcomes

Recognizing the evidence of the benefits associated with a lifelong commitment to exercise, the United States Department of Health and Human Services, as well as the AHA, have published physical activity guidelines for Americans. Current recommendations are that adults complete at least 150–300 min of moderate-intensity aerobic physical activity a week, 75–150 min of vigorous-intensity activity, or an equivalent of both [50]. In addition, muscle-strength training (e.g. weightlifting) should be completed twice per week. Generally, moderate-intensity exercise includes activity where an individual can talk in a few sentences, but not sing; vigorous-intensity exercise includes activity where a person can only say a few words at a time before having to take a breath [.

The Canadian 24-h movement guidelines have also been broadly adapted across many countries [51 ]. These guidelines encompass a combination of physical activity and sedentary behavior. Physical activity guidelines are similar to AHA guidelines as described, but also emphasize thatindividuals should engage in light physical activities for several hours per day. In addition, the Canadian guidelines emphasize the importance of limiting sedentary activity to less than eight hours per day, with fewer than three hours of screen time [51].

It was recently reported that adherence to the AHA guidelines is associated with meaningful improvements in survival. In a large analysis of adults (*N* = 116,221) from two large prospective cohorts (Nurses’ Health Study and the Health Professionals Follow-up Study) followed for 30 years, the hazard ratio comparing individuals meeting the vigorous-intensity guideline vs. not meeting this guideline was 0.81 (95% CI, 0.76–0.87) for all-cause mortality, 0.69 (95% CI, 0.60–0.78) for CVD and 0.85 (95% CI, 0.79–0.92) for non-CVD mortality [34]. Similarly, individuals meeting the guidelines for moderate-intensity exercise had reductions in all-cause mortality and CVD and non-CVD mortality compared to individuals who did not meet this exercise guideline [34]. Interestingly, higher levels of exercise, ie - exceeding the guidelines, was not associated with a further improvement in survival, indicating that as exercise intensity increases, the benefit plateaus [34].

**Figure 4.**
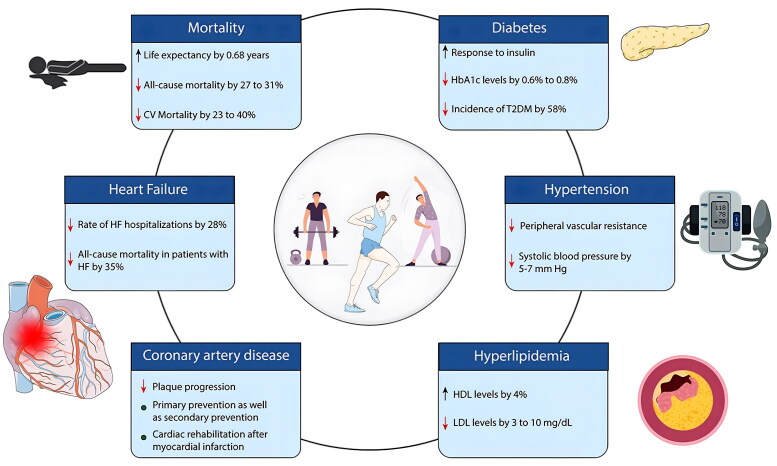
Impact regular exercise has on various cardiovascular outcomes. Image borrowed from isath et al. [62] with permission.

The concept of a “weekend warrior” has gained popularity and it appears that benefits of exercise are independent of the timing of exercise throughout the week. In a retrospective analysis of the United Kingdom Biobank, individuals (*N* = 89,573) exercising routinely with accelerometers were stratified into one of three groups: active weekend warrior, defined as individuals who achieved weekly exercise guidelines in 1–2 days throughout the week; active regular (achieving the guidelines throughout the course of the week); or inactive, defined as not meeting the guidelines [52]. Authors found that both activity patterns were associated with similar reductions in risk of incident atrial fibrillation, MI, heart failure and stroke [52]. In a similar study, individuals (*N* = 350,978) enrolled in the United States National Health Interview Survey were stratified into three groups: Physically inactive (not meeting the exercise guidelines), weekend warrior (meeting exercise guidelines in 1–2 days), or regularly active (meeting exercise guidelines over 3 or more days) [53]. Compared to the physically inactive group, the hazard ratio for all-cause mortality was 0.92 (95% CI, 0.83–1.02) for weekend warriors and 0.85 (95% CI, 0.83–0.88) for regularly active participants [53]. For the same amount of physical activity, the weekend warriors had similar all-cause mortality rates as the regular exercisers. Similar results were observed for cause-specific mortality, including CVD and cancer mortality [53]. Thus, large bodies of evidence support adherence to current exercise guidelines and the benefits of exercise appear to extend to individuals regardless of when, throughout the course of the week, exercise is undertaken. These positive benefits of exercise on cardiovascular outcomes and mortality have been shown to be independent of many factors including sex [54,55], age [56–58], and comorbidities [59–61]. Figure 4 outlines some of these health benefits associated with adherence to a routine exercise program [62].

While adherence to exercise guidelines improves the personal health of individuals, there are implications on a societal level as well. As of 2016 federal data, only 26% of men and 19% of women report performing the recommended amount of weekly activity [63]. An estimated $117 billion in annual healthcare costs in the US and around 10% of premature deaths are associated with inadequate physical activity [24]. These statistics underscore the importance of adherence to an exercise training program. Various sectors of the National Physical Activity Plan with proposed interventions to help patients engage in exercise are noted in Table 1 [50].

**Table 1.** Sectors in the 2016 National physical activity Plan and their potential role in supporting physical activity. Borrowed from piercy et al. [50] with permission.

National physical activity plan sector	Role
Business and industry	Employers can encourage workers to be physically active. They can provide access to facilities and encourage their use through outreach activities. Businesses can consider access to opportunities for active transportation and public transit when selecting new locations.
Community, recreation, fitness, and parks	This sector plays a leading role in providing access to places for active recreation, such as playgrounds, hiking and biking trails, senior centers, sports fields, and swimming pools. This sector can also provide access to exercise programs and equipment for a broad range of people, including underserved populations and people with disabilities.
Education	This sector can take a lead role in providing opportunities for age-appropriate physical activity in all educational settings. Opportunities include offering physical education, after-school sports, and public access to school facilities during after-school hours, and expanded intramural sports and campus recreation opportunities.
Faith-based settings	Faith-based organizations can be important partners in providing access to places for physical activity and promotion through outreach activities that can be tailored for diverse faith-based groups.
Health care	Health care professionals can assess, counsel, and advise patients on physical activity and how to do it safely. Health care systems can partner with other sectors to promote access to community-based physical activity programs.
Mass media	Media outlets can provide easy-to-understand messages about the health benefits of physical activity as part of community promotion efforts. Messages can also provide information about facilities or outlets where individuals can be active.
Public health	Public health departments can monitor community progress in providing places and opportunities to be physically active and track changes in the proportion of the population meeting the *Physical Activity Guidelines for Americans*. They can also take the lead in setting objectives and coordinating activities among sectors. Public health departments and organizations can disseminate appropriate messages and information to the public about physical activity.
Sports	This sector can provide organized opportunities for people to be active. Youth sports can expose children and adolescents to a variety of age-appropriate activities that can set the basis for a lifetime of activity. Sports organizations can also ensure that sports programs are conducted in a manner that minimizes risk of injuries.
Transportation, land use, and community design	This sector plays a lead role in designing and implementing options that provide areas for safe walking, bicycling, and wheelchair walking. Public transit systems also promote walking, as people typically walk to and from transit stops. Community planners and designers can implement design principles to create communities with activity-friendly routes to everyday destinations for people of all ages and abilities. They can also help create or improve access to places for physical activity, such as parks and other green spaces.

## Limitations

There are some limitations associated with this review. Most available data are derived from patients in the United States, which may limit the generalizability of findings to athletes from other regions with different healthcare systems, genetic backgrounds, and training environments. Additionally, access to cardiovascular care and research studies across countries could impact the applicability of US-based recommendations to other populations. Also, some studies in this field rely on retrospective analyses or observational designs that may introduce biases. Heterogeneity of study populations, including differences in sport type, training intensity, competition level, and other factors can complicate interpretation of results as well. Future research in this field should aim to incorporate more diverse exercises and employ rigorous prospective study design to help advance this field.

## Conclusion

Routine adherence to exercise training is associated with many benefits, including reductions in risk of CVD, stroke, all-cause mortality, and cause-specific mortality. Exercise is also associated with improvements in CVD risk factors, including cholesterol profile, blood pressure, glucose control and insulin sensitivity, as well as endothelial function. Guidelines have been established by major health agencies including the US Department of Health and Human Services, as well as the AHA, to provide specific recommendations on the “dose” of exercise that individuals should engage in.

## Data Availability

Data sharing is not applicable to this article as no new data were created or analyzed in this study.
